# Michigan State Board of Health

**Published:** 1878-12

**Authors:** 


### MICHIGAN STATE BOARD OF HEALTH.

*(Reported for the Chicago Medical Journal & Examiner.)*

The regular quarterly meeting was held in the office of the Secretary of State, at Lansing, Tuesday, Oct. 8, all the members being present, as follows: R. C. Kedzie, M. D., Prest., of Lansing; H. O. Hitchcock, M. D., of Kalamazoo; Henry F. Lyster, M. D., of Detroit; Hon. LeRoy Parker, of Flint; Rev. D. C. Jacokes, of Pontiac; and Henry B. Baker, secretary, of Lansing.

Rev. D. C. Jacokes, committee on buildings, heating, ventilation, etc, mentioned experiments in progress with regard to a plan of ventilation applicable to houses already built, on which he should be able to report at the next meeting. He had given illustrated lectures in a town in this State, and secured the construction of a chimney with ventilating flue, as recommended by Dr. Kedzie in the first annual report of the board, in a church where patent ventilating machines had failed; and the same plan would also be tried in private houses in that town. In this way he hoped to secure practical methods of ventilation "for the million." He had visited a house so constructed and shaded that scarcely a ray of direct sunlight could enter, and in which seven of eight children had died. He had visited, by authority of the board, several large ecclesiastical gatherings, and spoken on subjects connected with the work of this board, and through them had distributed important documents to all parts of the State.

Dr. Lyster, committee on climate, presented the introduction of a paper on the topography and climate of Michigan, illustrated with diagrams showing isothermal lines, rainfall, elevation of localities, distribution of woodland, frequency of storms, etc. It is to be completed for publication in the annual report.

Hon. LeRoy Parker, committee on legislation for public health, read a paper on coroners and coroners' juries, showing the former dignity of the office of coroner, and stating that the jury were originally summoned principally as witnesses. He traced the growth of the system of coroners' juries, showing the origin of some abuses and the extent to which the system has been degraded, and clearly exhibited the need for expert medical ability on the part of the coroner in tracing to their causes deaths from violence and accident, and for expert legal ability in dealing with cases of homicide, suicide and murder. He recommended that the coroner's jury as now constituted be abolished, as has already been done in Massachusetts; and that efforts be made to secure as coroners men of expert medical and legal ability. He stated that the system now in force in Massachusetts was considered far superior to the old system.

Dr. Lyster spoke of some of the evils of the present system, and the importance and need of the proposed reform.

Dr. Baker read a report of public health subjects in the proceedings of the Michigan State Medical Society, at its meeting in Lansing in May last, which was ordered published in the annual report.

The secretary reported further correspondence relative to the sanitary convention proposed to be held at Coldwater in February, 1879; also, correspondence with makers of sanitary appliances, relative to exhibiting their inventions at these conventions. He exhibited Wheeler's disinfector (invented by W. F. Wheeler, 119 S. Fourth st., Phila., Pa.) for rendering the water supplied to urinals, water-closets, etc., itself a disinfectant, by means of carbolic soap; also, Houghton's reversible charcoal water-filter, designed to be attached to water-pipes at the point of delivery, which had been obtained for the same purpose from the agent, E. C. Houghton, 75 Devonshire street, Boston. The secretary presented the usual report of work in the office during the last quarter. Circulars have been sent to the clerks of cities and villages who have not made the return of health officers as required by law; copies of the vital statistics of Michigan and of the last report of the State Board of Health have been distributed to sanitarians, sanitary exchanges of the board, and others engaged in sanitary work in the State; 20,000 copies of the document on restriction and prevention of diphtheria have been printed and are being distributed; replies of correspondents relative to diseases of 1877 have been compiled for the annual report; other manuscripts have been prepared for the report of the board for 1878, and the president's address and a few other articles have been printed. The annual inventory of property purchased, issued, used, and on hand, has been made. The meteorological registers supplied by the observers of the board for the year 1877 have been compiled as a basis for an article on the "Principal Meteorological Conditions of the Year." The report concerning the property of the board showed an addition to the library by gift, exchange or purchase, of 258 volumes, mostly bound.

A motion was passed directing a complete set of meteorological observations to be taken in the office of the secretary.

A motion was also passed directing that the document upon the restriction and prevention of diphtheria, recently issued by the board, be electrotyped for use by local boards of health and others interested in the restriction of the disease.

Dr. Lyster presented a communication from a dentist of Detroit, relative to the preservation and care of the teeth, which was read and discussed.

The secretary presented a communication from Dr. O. Marshall, of North Lansing, relative to opium-eating and the opium habit, which included a summary of replies by 96 prominent physicians in Michigan to a circular of inquiry as to the number of opium and morphine-eaters of both sexes in their localities. There were reported from these places, mostly villages and smaller cities, 1,313 habitual eaters of the drug. It also included a statement from the United States treasury department, showing that there have been imported during the 27 years from 1850 to 1877, 5,299,774 lbs. of opium, valued at $26,142,085, besides 22,565 oz. of morphine, valued at $73,433, imported during the 17 years from 1861 to 1877 ; also, showing that the imports for the ten years ending with 1877 exceed by 2,057,080 lbs., or nearly 200 per cent., the imports for the ten years ending with 1859. In addition, it is believed that there has been at least 10 per cent. of the amount above stated smuggled into the country. The estimates for Michigan based on these replies and the statements of a wholesale drug-house in Detroit show the consumption of opium to be very large.

The secretary read a communication from Dr. Topping, of Dewitt, based on recent experiences in an epidemic of diphtheria, in which he reversed a former report that he had seen no evidences of the contagiousness of the disease, having lately traced about 70 cases to one first case. Also, two communications from Dr. J. S. Caulkins, of Thornville, relative to the period of incubation in diphtheria, based on a study of nine cases in one family, six of which were fatal, and eight cases in another family, four of which were fatal.

A resolution was adopted, directing the committee on legislation to prepare a memorial to the legislature in favor of a topographical survey of the whole State for sanitary purposes, to be instituted at once and carried forward to its earliest completion.

Dr. Baker was appointed a committee to confer with the representatives of the American Public Health Association, American Social Science Association, and others, relative to devising some method for uniform registration of births, marriages and deaths, throughout the United States.

After the auditing of bills, and a large amount of routine business, the board adjourned to meet Jan. 7, 1879.

